# Aortic Root Dilatation in Taiwanese Patients with Mucopolysaccharidoses and the Long-Term Effects of Enzyme Replacement Therapy

**DOI:** 10.3390/diagnostics11010016

**Published:** 2020-12-23

**Authors:** Hsiang-Yu Lin, Ming-Ren Chen, Chung-Lin Lee, Shan-Miao Lin, Chung-Lieh Hung, Dau-Ming Niu, Tung-Ming Chang, Chih-Kuang Chuang, Shuan-Pei Lin

**Affiliations:** 1 Department of Medicine, MacKay Medical College, New Taipei City 25245, Taiwan; lxc46199@ms37.hinet.net (H.-Y.L.); mingren44@gmail.com (M.-R.C.); miao1029@gmail.com (S.-M.L.); jotaro3791@gmail.com (C.-L.H.); 2 Department of Pediatrics, MacKay Memorial Hospital, Taipei 10449, Taiwan; 3 Department of Medical Research, MacKay Memorial Hospital, New Taipei City 25160, Taiwan; 4 MacKay Junior College of Medicine, Nursing and Management, Taipei 11260, Taiwan; clampcage@yahoo.com.tw; 5 Department of Medical Research, China Medical University Hospital, China Medical University, Taichung 40402, Taiwan; 6 Department of Rare Disease Center, MacKay Memorial Hospital, Taipei 10449, Taiwan; 7 Department of Pediatrics, MacKay Memorial Hospital, Hsinchu 30071, Taiwan; 8 Institute of Clinical Medicine, National Yang-Ming University, Taipei 11221, Taiwan; dmniu1111@yahoo.com.tw; 9 Division of Cardiology, Department of Internal Medicine, MacKay Memorial Hospital, Taipei 10449, Taiwan; 10 Department of Pediatrics, Taipei Veterans General Hospital, Taipei 11217, Taiwan; 11 Department of Pediatric Neurology, Changhua Christian Children’s Hospital, Changhua 500, Taiwan; 128658@cch.org.tw; 12 School of Medicine, Kaohsiung Medical University, Kaohsiung 80708, Taiwan; 13 College of Medicine, Fu-Jen Catholic University, Taipei 24205, Taiwan; 14 Department of Infant and Child Care, National Taipei University of Nursing and Health Sciences, Taipei 11219, Taiwan

**Keywords:** aortic root dilatation, echocardiography, enzyme replacement therapy, mucopolysaccharidosis

## Abstract

Background: Cardiovascular abnormalities have been observed in patients with mucopolysaccharidosis (MPS) of any type, with the most documented abnormalities being valvular regurgitation and stenosis and cardiac hypertrophy. Only a few studies have focused on aortic root dilatation and the long-term effects of enzyme replacement therapy (ERT) in these patients. Methods: We reviewed echocardiograms of 125 Taiwanese MPS patients (age range, 0.1 to 19.1 years; 11 with MPS I, 49 with MPS II, 25 with MPS III, 29 with MPS IVA, and 11 with MPS VI). The aortic root diameter was measured at the sinus of Valsalva. Results: Aortic root dilatation (*z* score >2) was observed in 47% of the MPS patients, including 66% of MPS IV, 51% of MPS II, 45% of MPS VI, 28% of MPS III, and 27% of MPS I patients. The mean aortic root diameter *z* score was 2.14 (*n* = 125). The patients with MPS IV had the most severe aortic root dilatation with a mean aortic root diameter *z* score of 3.03, followed by MPS II (2.12), MPS VI (2.06), MPS III (1.68), and MPS I (1.03). The aortic root diameter *z* score was positively correlated with increasing age (*n* = 125, *p* < 0.01). For the patients with MPS II, III, and IV, aortic root diameter *z* score was also positively correlated with increasing age (*p* < 0.01). For 16 patients who had received ERT and had follow-up echocardiographic data (range 2.0–16.2 years), the mean aortic root diameter *z* score change was −0.46 compared to baseline (baseline 2.49 versus follow-up 2.03, *p* = 0.490). Conclusions: Aortic root dilatation was common in the patients with all types of MPS, with the most severe aortic root dilatation observed in those with MPS IV. The severity of aortic root dilatation worsened with increasing age, reinforcing the concept of the progressive nature of this disease. ERT for MPS appears to stabilize the progression of aortic root dilatation.

## 1. Introduction

The mucopolysaccharidoses (MPSs; OMIM 252700) are a group of lysosomal storage diseases resulting from deficiencies in specific lysosomal enzymes and are characterized by the sequential catabolism of glycosaminoglycans (GAGs) causing progressive substrate accumulation in various tissues and organs. Seven different types of MPS disorders (I, II, III, IV, VI, VII, and IX) with 11 specific lysosomal enzyme deficiencies have been described. The clinical signs and symptoms may manifest from early to late childhood or even in early adulthood according to the type and severity of MPS. The clinical presentations in these patients include coarse facial features, airway obstruction, cardiovascular dysfunction, pulmonary function impairment, hepatosplenomegaly, skeletal deformities (dysostosis multiplex), short stature, and developmental delay [1,2]. Recurrent respiratory infections, respiratory failure, and cardiac failure usually occur before 10 years of age in patients with the severe forms [3,4,5,6,7].

It is well known that cardiovascular dysfunction increases morbidity and mortality in these patients. However, the onset and extent of cardiovascular involvement differ for each MPS type [8,9,10]. Cardiac valve thickening, valvular regurgitation and stenosis, and cardiac hypertrophy are the most common cardiologic defects of MPS. GAG accumulation in the cardiac valves, myocardium, great vessels, and coronary arteries results in valvular defects and cardiomyopathy. Heart failure, coronary occlusion, and sudden death from arrhythmias are all cardiac causes of death [3,4,5,11].

The great vessels in MPS patients can be constricted or dilated by thickening of their walls. Thickening, disruption of elastin fibers, and GAG accumulation are histological abnormalities found in aortic walls. Aortic root dilatation is frequently observed in animal models of MPS I, leading to the effacement of the sinotubular ridge [8]. Some studies have reported that aortic root dilatation may appear in various types of MPS patients, especially in those with MPS IVA [12,13,14,15,16,17,18].

The principal treatments for MPS disorders include hematopoietic stem cell transplantation (HSCT) and enzyme replacement therapy (ERT). HSCT has been performed for MPS I, II, IVA, VI, and VII, showing therapeutic benefits in endurance, joint mobility, hepatosplenomegaly, growth, respiratory functions, and upper airway obstruction. ERT is presently available for MPS I, II, IVA, VI, and VII, and it has been shown to substantially reduce urinary GAG levels and significantly improve endurance, joint mobility, hepatosplenomegaly, lung function, and quality of life in these patients [19]. However, animal models of MPS have reported that ERT had little effect on lysosomal storage accumulation in aortic smooth muscle cells, and thus had limited impact on aortic root dilatation related to the disease [20,21]. To date, only a few reports have focused on aortic root dilatation and the long-term effects of ERT in MPS patients [15]. The purpose of this study was to investigate aortic root dilatation in Taiwanese patients with different types of MPS and evaluate the long-term effects of ERT.

## 2. Materials and Methods

### 2.1. Study Population

The medical records and echocardiograms of 125 Taiwanese patients with different types of MPS (85 males and 40 females; mean age, 8.1 ± 5.3 years; median age, 7.1 years; age range, 0.1 to 19.1 years; 11 patients with MPS I, 49 patients with MPS II, 25 patients with MPS III, 29 patients with MPS IVA, and 11 patients with MPS VI) were retrospectively reviewed at MacKay Memorial Hospital from August 1991 to June 2020. The diagnosis of the type of MPS was confirmed by a specific enzyme activity assay in serum, leukocytes, and/or skin fibroblasts; two-dimensional electrophoresis of urinary GAGs; and/or identification of the pathogenic mutations [22]. For the MPS II patients, the severe form was defined on the basis of the presence of cognitive impairment in comparison to the mild form without cognitive impairment. None of the patients received ERT or HSCT at baseline. We also reviewed and analyzed the aortic root diameters of 16 patients with 2.0 to 16.2 years of follow-up data who had received ERT. The patients’ ages at which ERT began ranged widely from 0.7 to 18.2 years. The MPS I patients received 0.58 mg/kg/week intravenous laronidase (Aldurazyme, Sanofi Genzyme, Boston, MA, USA), the MPS II patients received 0.5 mg/kg/week intravenous idursulfase (Elaprase, Shire Pharmaceutical Inc., Lexington, MA, USA), the MPS IVA patients received 2.0 mg/kg/week intravenous elosulfase alfa (Vimizim, BioMarin Pharmaceutical Inc., Novato, CA, USA), and the MPS VI patients received 1.0 mg/kg/week intravenous galsulfase (Naglazyme, BioMarin Pharmaceutical Inc.). Written informed consent was obtained from each patient or their parents or legal representative.

### 2.2. Data Collection

A Philips Sonos 5500/7500 ultrasound system (Andover, MA, USA) equipped with electronic transducers from 2 to 8 MHz was used to measure echocardiographic parameters. One experienced cardiologist (MRC) digitally stored and analyzed the data to minimize inter-observer variations. In this study, only measurements of aortic root diameter obtained at the sinus of Valsalva (SoV) in the echocardiograms were used. The aorta was measured on the sinus from the leading edge to leading edge. Aortic root diameter *z* scores adjusted for body surface area were determined as described by Colan et al. [23] and confirmed using the method described by Dallaire et al. [24]. The body surface area was calculated using the Haycock formula [25]. Aortic root dilatation was defined as an aortic root diameter *z* score >2.0.

### 2.3. Data Analysis and Statistics

Sex, age, height, weight, and body surface area at the time of echocardiographic evaluations were recorded for each patient. Descriptive statistics including means and standard deviations of all demographic values were calculated. The aortic root diameter *z* scores of each MPS type were compared and analyzed using two-way analysis of variance (ANOVA). Relationships between ages and aortic root diameter *z* scores were analyzed using Pearson’s correlation coefficient (*r*), and significance was assessed using Fisher’s *r–z* transformation. Two-tailed *p*-values were calculated. All statistical analyses were performed using SPSS version 11.5 (SPSS Inc., Chicago, IL, USA). Differences with *p* < 0.05 were considered to be statistically significant.

## 3. Results

**Figure 1:**
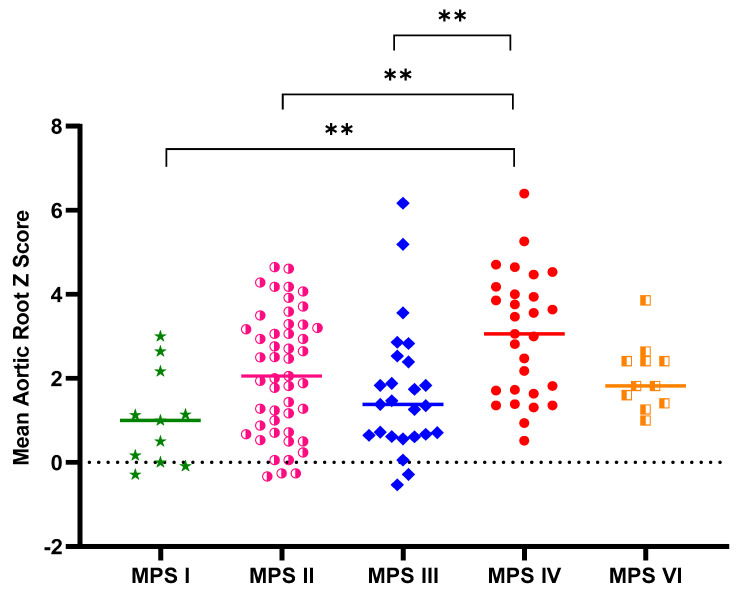
Aortic root diameter *z* score in the patients with different types of MPSs (*n* = 125) (** *p* < 0.01). There were significant differences in aortic root diameter *z* scores between those with MPS IV and MPS I, MPS IV, and MPS II, as well as MPS IV and MPS III (all *p* < 0.01).

**Figure 2:**
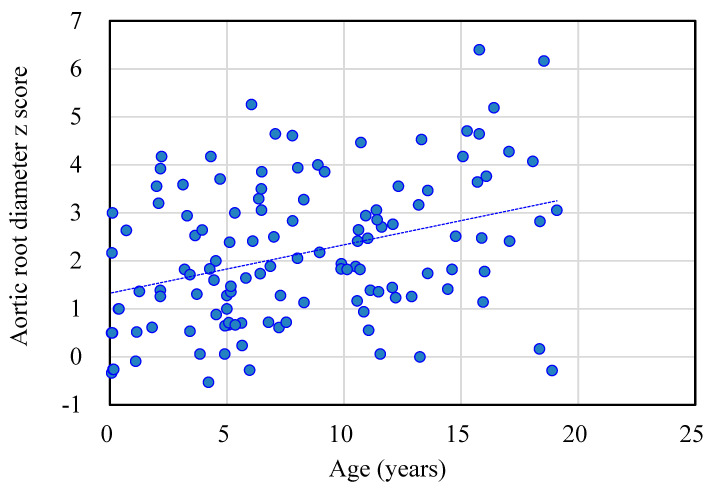
The aortic root diameter *z* score was positively correlated with age in the 125 patients with MPS (*r* = 0.353, *p* < 0.01). The dotted line represents the trendline.

**Figure 3:**
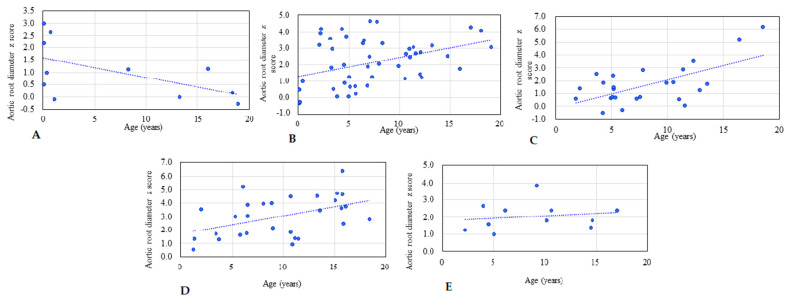
The relationships between aortic root diameter *z* score and age for each type of MPS. (**A**) MPS I (*n* = 11, *r* = −0.566, *p* < 0.05); (**B**) MPS II (*n* = 49, *r* = 0.394, *p* < 0.01); (**C**) MPS III (*n* = 25, *r* = 0.624, *p* < 0.01); (**D**) MPS IV (*n* = 29, *r* = 0.457, *p* < 0.01); (**E**) MPS VI (*n* = 11, *r* = 0.161, *p* > 0.05). The dotted line represents the trendline.

**Figure 4:**
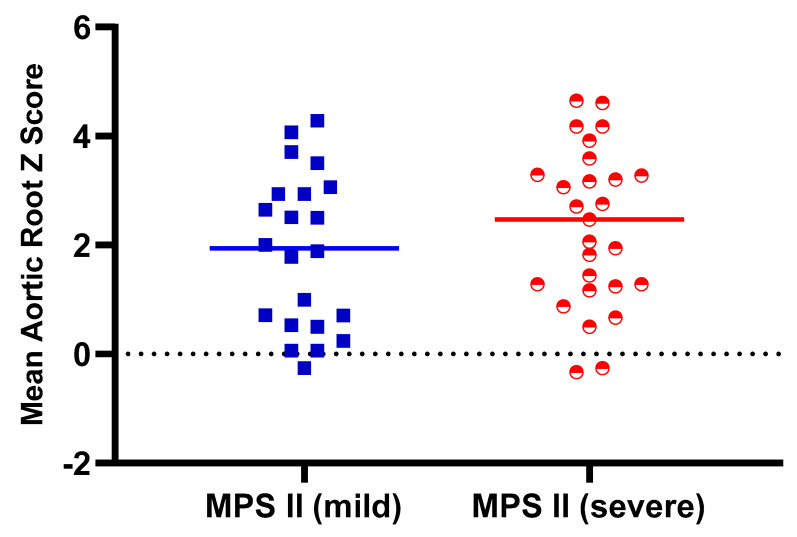
Aortic root diameter *z* score in the patients with the mild and severe forms of MPS II (*p* = 0.287).

**Figure 5:**
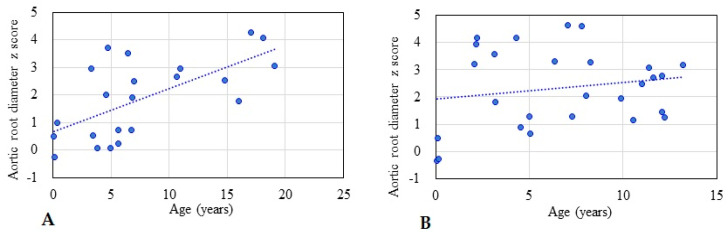
The relationships between aortic root diameter *z* score and age in the patients with the mild and severe forms of MPS II: (**A**) mild form (*n* = 22, *r* = 0.642, *p* < 0.01); (**B**) severe form (*n* = 27, *r* = 0.180, *p* > 0.05). The dotted line represents the trendline.

Table 1 shows the baseline demographic characteristics and echocardiographic aortic root diameter measurements of the 125 patients with different types of MPS. Aortic root dilatation (*z* score > 2) was observed in 47% of all patients, including 66% of those with MPS IV, 51% of those with MPS II, 45% of those with MPS VI, 28% of those with MPS III, and 27% of those with MPS I. The mean aortic root diameter *z* score was 2.14 (*n* = 125). The patients with MPS IV had the most severe aortic root dilatation (mean aortic root diameter z score of 3.03), followed by those with MPS II (2.12), MPS VI (2.06), MPS III (1.68), and MPS I (1.03). There were significant differences in aortic root diameter *z* scores between the patients with MPS IV and MPS I, MPS IV and MPS II, as well as MPS IV and MPS III (all *p* < 0.01) (Figure 1). The aortic root diameter *z* score was positively correlated with increasing age (*n* = 125, *p* < 0.01) (Figure 2). For the patients with MPS II, III, and IV, aortic root diameter *z* score was also positively correlated with increasing age (*p* < 0.01). In the patients with MPS I, the aortic root diameter *z* score was negatively correlated with increasing age (*p* < 0.05). However, there was no significant correlation between aortic root diameter *z* score and age in the patients with MPS VI (Figure 3). Table 2 shows the baseline demographic characteristics and echocardiographic aortic root diameter measurements of the 49 patients with MPS II subdivided into the mild form (*n* = 22) and severe form (*n* = 27). Aortic root dilatation (*z* score > 2) was observed in 45% of those with the mild form and in 56% of those with the severe form. The mean aortic root diameter *z* score was 1.88 for those with the mild form and 2.32 for those with the severe form (*p* > 0.05) (Figure 4). For the 22 patients with the mild form of MPS II, aortic root diameter *z* score was also positively correlated with increasing age (*p* < 0.01). However, for the 27 patients with the severe form of MPS II, there was no significant correlation between aortic root diameter *z* score and increasing age (Figure 5). For 16 patients (3 with MPS I, 5 with MPS II, 5 with MPS IVA, and 3 with MPS VI) who had received ERT and had follow-up echocardiographic data (range 2.0–16.2 years), 10 had improvements in aortic root diameter *z* score after ERT compared to baseline, with a mean change of −0.45 (baseline 2.67 versus follow-up 2.22, *p* = 0.501). Eleven patients had aortic root dilatation (*z* score > 2) at baseline compared to 8 patients who had aortic root dilatation after ERT (Table 3). ERT for MPS patients appeared to stabilize the progression of aortic root dilatation.

## 4. Discussion

To the best of our knowledge, this is the largest cohort to delineate aortic root dilatation and long-term effects of ERT in patients with different types of MPS in a single population. Aortic root dilatation in MPS indicates arterial elastin dysfunction [9]. Our results demonstrated a high prevalence of aortic root dilatation in all types of MPSs (47%), which is consistent with previous studies by Bolourchi et al. [14] (35%, *n* = 34, MPS I–VII), Poswar et al. [15] (39%, *n* = 69, MPS I, II, IVA, and VI), and Wang et al. [9] (56%, *n* = 9, MPS IVA). The severity of aortic root dilatation worsened with increasing age in our patients (*n* = 125, *p* < 0.01), reinforcing the concept of the progressive nature of this disease. In addition, we observed that the most severe aortic root dilatation occurred in the patients with MPS IV, which is consistent with previous studies [14,15].

Aortic root dilatation typically evolves through cystic medial degeneration and migration of smooth muscle cells from the tunica media to the tunica intima with age [26]. Animal model studies of MPS I mice [27] and MPS I and MPS VII dogs [20] have indicated that aortic dilatation most likely results from degradation of elastin by cathepsin S and matrix metalloproteinase-12. Dilatation of the ascending aorta and impairment of aortic elasticity have been described in attenuated MPS I [28]. The cause could be through the downstream effects of GAGs on the tropoelastin assembly, leading to fragmented elastic fibers in the aorta [29]. In our study, aortic root dilatation was found most frequently in the patients with MPS IV (66%), followed by those with MPS II (51%), MPS VI (45%), MPS III (28%), and MPS I (27%). The mean aortic root diameter *z* score was 2.14 (*n* = 125). The patients with MPS IV had the most severe aortic root dilatation with a mean aortic root diameter *z* score of 3.03, followed by those with MPS II (2.12), MPS VI (2.06), MPS III (1.68), and MPS I (1.03). The aortic root diameter *z* score was positively correlated with increasing age (*n* = 125). For the patients with MPS II, III, and IV, aortic root diameter *z* score was also positively correlated with increasing age, which is compatible with the progressive nature of MPS. However, in the patients with MPS I, aortic root diameter *z* score was negatively correlated with increasing age (*p* < 0.05). A possible explanation for this finding is that patients with the severe form may have a shorter life expectancy, and thus older patients with MPS I mostly have the attenuated form with a less significant involvement of the aorta. Our results are consistent with those of a previous study [15].

MPS II can be classified into severe and mild forms according to whether or not the patients have impaired intellectual disability. Patients with the neuronopathic form (severe form) tend to have more prominent somatic manifestations [30]. In our patients with MPS II, aortic root dilatation was observed in 45% of those with the mild form and in 56% of those with the severe form (mean aortic root diameter *z* score 1.88 versus 2.32, respectively), which is similar to a previous study [15]. In addition, in the 22 patients with the mild form of MPS II in our study, the aortic root diameter *z* score was also positively correlated with increasing age, which is consistent with the progressive nature of this disease.

Aortic dissection can occur in patients with connective tissue disorders, including Marfan syndrome, Ehlers–Danlos syndrome, and Turner syndrome [31,32]. While patients with MPS have not been demonstrated to be at an increased risk for this uncommon life-threatening condition, pooling of GAGs is recognized to be a distinguishing histopathological characteristic of thoracic aortic aneurysm and dissection [33]. The clinical importance of identifying aortic root dilatation is that it may develop into aortic dissection [34]. Although there is currently no consensus for when to perform aortic surgery according to aortic root diameter *z* score value, it is generally presumed that a value of <4.0 does not indicate an impending risk of aortic dissection [31]. None of the patients in the studies by Bolourchi et al. [14] and Poswar et al. [15] had aortic dissection, and none of the 15 MPS patients (12%) with aortic root diameter *z* scores >4.0 (6 MPS II (12%), 2 MPS III (8%), and 7 MPS IV (24%)) in the current study had aortic dissections. Therefore, routine screening for this potentially crucial factor should be incorporated into the multidisciplinary care for these patients. While no patient with MPS has yet been reported to have aortic dissection, the increase in life span of these patients who receive ERT or HSCT warrants its identification to allow for prompt and effective medical interventions if symptoms occur.

It is currently unclear whether ERT can prevent or treat aortic root dilatation in MPS patients [13,14,15]. In the current study, of the 16 patients who had received ERT and had 2.0–16.2 years of follow-up echocardiographic data, 10 had improvements in aortic root diameter *z* score compared to baseline. The mean aortic root diameter *z* score change was -0.45, and 11 patients had aortic root dilatation at baseline compared with 8 patients after ERT. Since the severity of aortic root dilatation worsened with increasing age in this cohort, ERT for MPS appeared to stabilize the progression of aortic root dilatation.

Studies of MPS I mouse models have demonstrated that angiotensin receptor blockade therapy with losartan can prevent aortic root dilatation. Moreover, this medication could ameliorate both the cardiac and craniofacial manifestations and could potentially be given to MPS patients under ERT [35,36].

In the recent decade, the increasing clinical awareness of MPS disorders and increased ability to make a confirmative diagnosis has made an earlier diagnosis possible. Due to the progressive nature of MPS, the timely initiation of ERT, HSCT, as well as other appropriate medical interventions before the appearance of irreversible organ damage may lead to a better clinical outcome. As a result, making an early diagnosis through high-risk population screening programs or newborn screening programs is very important [37,38,39,40].

### Limitations

As a retrospective and uncontrolled study, there was no healthy control group, and thus we could not compare aortic root measurements between the patients and healthy controls. In addition, since MPS patients tend to have a disproportionately short stature, their *z* scores for aortic root measurements, which are normalized to body surface area, may have been overestimated. We used reference values from a Caucasian population due to the lack of reference values from an Asian population. Moreover, the relatively small number of patients with each type of MPS reflects the rare nature of this genetic disorder. Furthermore, both the age range (0.1–19.1 years) and degree of disease severity varied widely. Due to the limitation of the study design, those patients received ERT for different lengths of time, and the number of ERT patients (*n* = 16) was too low to draw statistically sound conclusions. As a result, studies with larger cohorts and longer follow-up periods are warranted.

## 5. Conclusions

Aortic root dilatation was common in patients with all types of MPSs in this study, with the most severe aortic root dilatation observed in those with MPS IV. The severity of aortic root dilatation worsened with increasing age, reinforcing the concept of the progressive nature of this disease. ERT for MPS patients appeared to stabilize the progression of aortic root dilatation. Thus, the routine screening for this potentially crucial factor should be incorporated into the multidisciplinary care for these patients. Due to the progressive nature of MPS, making an early diagnosis and initiating the most appropriate medications before the appearance of irreversible organ damage is very important.

## Figures and Tables

**Table 1:** Demographic characteristics and aortic root diameter measurements among patients with different types of mucopolysaccharidoses (MPSs) (*n* = 125).

	MPS I	MPS II	MPS III	MPS IV	MPS VI	All
*n*	11	49	25	29	11	125
Gender (M/F)	5/6	49/0	13/12	13/16	5/6	85/40
Age (years)	7.0 (8.1)	7.2 (5.0)	8.1 (4.4)	9.8 (5.1)	8.9 (5.0)	8.1 (5.3)
Age range (years)	0.1–18.9	0.1–19.1	1.8–18.5	1.1–18.4	2.2–17.1	0.1–19.1
Height *z* score	−2.59 (3.96)	−1.81 (2.77)	−0.73 (1.75)	−6.16 (3.43)	−5.13 (3.15)	−2.96 (3.56)
Weight *z* score	−0.92 (1.14)	0.04 (1.94)	−0.13 (1.51)	−2.09 (1.13)	−1.78 (1.10)	−0.73 (1.80)
BSA (m^2^)	0.65 (0.41)	0.78 (0.25)	0.92 (0.24)	0.69 (0.20)	0.69 (0.10)	0.77 (0.26)
AoD (cm)	1.67 (0.39)	2.10 (0.47)	2.19 (0.44)	2.11 (0.31)	1.99 (0.16)	2.07 (0.42)
AoD *z* score	1.03 (1.13)	2.12 (1.43)	1.68 (1.57)	3.03 (1.48)	2.06 (0.80)	2.14 (1.50)
AoD *z* score > 2, *n* (%)	3 (27%)	25 (51%)	7 (28%)	19 (66%)	5 (45%)	59 (47%)
*r* value (AoD *z* score versus age)	−0.566	0.394	0.624	0.457	0.161	0.353
*p*-value	*p* < 0.05	*p* < 0.01	*p* < 0.01	*p* < 0.01	*p* > 0.05	*p* < 0.01

Data are mean (standard deviation). MPS, mucopolysaccharidosis; M/F: male/female; BSA: body surface area; AoD, aortic root diameter.

**Table 2:** Demographic characteristics and aortic root diameter measurements in patients with the mild form and severe form of MPS II (*n* = 49).

Covariate	MPS II (Mild)	MPS II (Severe)
*n*	22	27
Gender (M/F)	22/0	27/0
Age (years)	7.7 (5.9)	6.7 (4.2)
Age range (years)	0.1–19.1	0.1–13.2
Height *z* score	−2.03 (3.23)	−1.63 (2.38)
Weight *z* score	0.06 (1.62)	0.03 (2.19)
BSA (m^2^)	0.82 (0.29)	0.75 (0.21)
AoD (cm)	2.10 (0.54)	2.10 (0.42)
AoD *z* score	1.88 (1.43)	2.32 (1.44)
AoD *z* score > 2, *n* (%)	10 (45%)	15 (56%)
*r* value (AoD *z* score versus age)	0.642	0.180
*p*-value	*p* < 0.01	*p* > 0.05

Data are mean (standard deviation). MPS, mucopolysaccharidosis; M/F: male/female; BSA: body surface area; AoD, aortic root diameter.

**Table 3:** Baseline and follow-up data of aortic root diameter of 16 patients with MPS who received enzyme replacement therapy for 2.0–16.2 years.

No.	MPS Type	Gender	Age at Baseline (Years)	Age at Follow-Up (Years)	ERT Duration (Years)	AoD (cm)		AoD (*z* Score)		Change (*z* Score)
						Baseline	Follow-Up	Baseline	Follow-Up	
1	I (H/S)	F	0.7	7.7	7.0	1.70	1.90	2.60	0.60	−2.00
2	I (H/S)	F	1.1	6.9	5.8	1.40	2.00	−0.10	1.20	1.30
3	I (H/S)	M	18.2	20.2	2.0	1.63	1.88	−2.00	−0.61	1.39
4	II (M)	M	13.2	25.7	12.4	2.28	2.50	1.12	0.52	−0.60
5	II (M)	M	14.8	23.3	8.5	2.54	3.10	2.51	2.74	0.23
6	II (M)	M	15.4	17.7	2.2	2.85	2.14	4.47	0.29	−4.18
7	II (M)	M	17.5	29.8	12.3	2.80	2.90	3.00	2.00	−1.00
8	II (M)	M	18.1	26.6	8.6	3.46	3.70	4.07	4.06	−0.01
9	IVA	M	1.1	7.4	6.3	1.56	2.40	0.52	4.18	3.66
10	IVA	M	2.3	6.6	4.3	1.64	1.80	0.92	1.20	0.28
11	IVA	M	7.2	10.5	3.3	2.08	2.30	2.29	2.39	0.10
12	IVA	F	14.8	19.0	4.2	2.42	2.40	4.29	3.59	−0.70
13	IVA	F	16.8	23.2	6.4	2.80	2.60	5.94	3.39	−2.55
14	VI	M	7.6	21.6	14.0	2.06	2.20	3.57	1.83	−1.74
15	VI	M	8.3	22.4	14.1	2.38	2.80	5.07	4.18	−0.89
16	VI	M	11.7	27.9	16.2	2.67	2.70	4.44	3.94	−0.50
Mean			10.6	18.5	8.0	2.27	2.46	2.67	2.22	−0.45

MPS, mucopolysaccharidosis; ERT, enzyme replacement therapy; AoD, aortic root diameter; H/S: Hurler–Scheie; MPS II (M), mild form; F, female; M, male. *p*-value: *p* = 0.501.
